# Hair-Template Confinement Assembly of Nanomaterials Enables a Robust Single-Hair Surface-Enhanced Raman Spectrocopy Platform for Trace Analysis

**DOI:** 10.3390/nano15201557

**Published:** 2025-10-13

**Authors:** Miao Qin, Siyu Chen, Tao Xie, Mingwen Ma, Cong Wang

**Affiliations:** 1 Key Laboratory of Spin Electron and Nanomaterials of Anhui Higher Education Institutes, School of Chemistry and Chemical Engineering, Suzhou University, Suzhou 234000, China; mamingwen163@163.com (M.M.); congwang@ahszu.edu.cn (C.W.); 2 Information Materials and Intelligent Sensing Laboratory of Anhui Province, Anhui University, Hefei 230039, China; 23068@ahu.edu.cn; 3 Key Laboratory of Conservation and Utilization of Dabie Mountain Characteristic Biological Resources, West Anhui University, Lu’an 237012, China; xt2020@mail.ustc.edu.cn

**Keywords:** surface-enhanced Raman spectroscopy, hair-templated plasmonic nanoarrays, gold nanorods, capillary-evaporation-driven assembly, p-phenylenediamine

## Abstract

Surface-enhanced Raman spectroscopy (SERS) enables ultra-sensitive molecular detection and has broad analytical and biomedical applications; recent advances focus on high-performance substrates and innovative detection strategies. However, achieving controllable and reproducible substrate fabrication—particularly using natural templates such as hair—remains challenging, limiting SERS application in trace analysis and on-site detection. This study developed a single-hair in situ SERS platform using a natural hair template. Confinement within hair cuticle grooves and capillary-evaporation assembly enables dense arrangement of cetyltrimethylammonium bromide-coated Au nanorods and polyvinylpyrrolidone-coated Au nanoparticles, forming uniform plasmonic nanoarrays. Spectroscopy and microscopy analyses confirmed the regular alignment of nanostructures along the hair axis with denser packing at the edges. The platform detected crystal violet at 10^−9^ M, yielding clear signals, negligible background, and stable peaks after repeated washing. For p-phenylenediamine, enhancement was observed down to 10^−6^ M. On the platform, a concentration-dependent response appeared within 10^−3^–10^−5^ M, with spatial Raman imaging along the hair axis. Capillary-evaporation coupling and interfacial wettability facilitated solute enrichment from larger to smaller gap hotspots, improving signal-to-noise ratio and reproducibility. This portable, low-cost, and scalable method supports rapid on-site screening in complex matrixes, offering a general strategy for hotspot engineering and programmable assembly on natural templates.

## 1. Introduction

Since its discovery in the 1970s [1], surface-enhanced Raman spectroscopy (SERS) has become one of the most influential techniques in molecular analysis [2]. SERS relies on localized surface plasmon resonance (LSPR) on nanostructured surfaces and molecular chemical enhancement mechanisms, enabling ultra-sensitive detection of trace molecules [3]. Compared with traditional analytical methods, SERS offers extremely high sensitivity and detailed molecular fingerprint information, while also demonstrating broad applicability in chemical analysis, biomedicine, and environmental monitoring owing to its simple sample preparation and rapid detection [4,5]. In recent years, research in the field of SERS has concentrated on developing high-performance substrates and exploring innovative detection strategies [6,7,8]. Noble metal nanomaterials, such as gold and silver nanoparticles, are preferred substrates for SERS because of their excellent plasmonic properties. By tuning their size, morphology, and assembly methods, various SERS substrates with high enhancement, uniformity, and stability—such as nanorods, nanostars, and nanoflowers—have been fabricated [9,10]. Furthermore, the emergence of three-dimensional assemblies, ordered arrays, and flexible substrates has considerably expanded the application scope of SERS. In terms of detection strategies, methods such as SERS tags, imaging, and single-molecule detection continue to advance, promoting applications of SERS in frontier fields including life sciences and clinical diagnostics [11]. Despite these advances in SERS technology, challenges remain in achieving controllable and reproducible substrate fabrication, suppressing interference during detection, and enabling intelligent data analysis. Future priorities include developing low-cost, high-performance substrates suitable for large-scale production and implementing automated, intelligent detection methods to support the practical application of SERS technology.

Hair, as a natural biological template, offers distinct advantages for controllable assembly and SERS detection because nanomaterial performance depends not only on composition and size but also on microstructure and surface chemistry, and achieving structural regulation with functional optimization remains a central challenge in nanoscience [12]. The confined assembly strategy leverages physical or chemical confinement to induce oriented growth of nanomaterials in specific regions. Owing to its unique microstructure and abundant surface functional groups (amino, carboxyl, thiol), hair enables specific interactions with metallic nanomaterials, thereby supporting controlled assembly, functional modification, and the fabrication of SERS-active layers with precisely regulated density, arrangement, and configuration [12,13,14,15,15]. Compared with traditional inorganic or polymer substrates, hair-based substrates are more flexible, accessible, and inexpensive, making them particularly suitable for rapid on-site detection and trace analysis [15]. Assembling metallic nanoparticles on hair templates enables highly sensitive detection of target molecules, including environmental pollutants and drug residues, demonstrating strong potential for real-sample analysis [16,17].

Beyond serving as a SERS substrate, hair functions as a distinctive sampling medium and information carrier. Its long growth cycle and ability to accumulate exogenous substances allow reconstruction of long-term exposure histories. Related research has achieved sensitive detection of exogenous drugs and heavy metals, offering new avenues for public safety and health monitoring [18,19]. Integration of hair-based SERS with mass spectrometry and chromatography can further enhance the selectivity and accuracy of trace component detection in complex matrixes [20]. More broadly, as a template with rich functional groups and unique microarchitecture, hair promotes selective deposition and ordered arrangement of nanomaterials to produce nanocomposites with structural stability and diverse functionalities. The confined assembly strategy is extensible to other natural or synthetic templates to create hierarchical, multifunctional materials and to advance intelligent nanodevices and multimodal analytical platforms [21,22]. Despite these merits, biological templates face inherent challenges, including inter-individual variability, gradual biochemical degradation, and surface heterogeneity. Our confined-assembly approach alleviates part of these issues by (i) electrostatic guidance that is less sensitive to moderate charge fluctuation and (ii) capillary-driven self-limiting film formation; nonetheless, long-term environmental stability and universal applicability across chemically treated hairs warrant further study.

In this study, hair served as a natural template, and a confined assembly strategy was employed in situ to construct nanostructured arrays on the hair surface, resulting in a novel single-hair visualized Dynamic-SERS (D-SERS) detection method. By assembling gold nanoparticles in situ, capillary action and electrostatic interactions were harnessed to achieve oriented arrangement and uniform distribution on the hair surface, markedly improving the reproducibility and stability of the SERS substrate. This method not only expands the range of building blocks for hair-based SERS substrates but also provides a new approach for detecting transient residues of contaminants on hair surfaces. The fabricated hair SERS substrate shows strong potential for trace analysis in life and health sciences and could offer robust technical support for rapid on-site detection of real samples.

## 2. Materials and Methods

Cetyltrimethylammonium bromide (CTAB), chloroauric acid (HAuCl_4_), sulfuric acid (H_2_SO_4_), sodium borohydride (NaBH_4_), sodium hydroxide (NaOH), silver nitrate (AgNO_3_), nitric acid (HNO_3_), trisodium citrate (C_6_H_5_Na_3_O_7_), crystal violet (CV), polyvinylpyrrolidone (PVP), hydrogen peroxide (H_2_O_2_), ascorbic acid (AA), dodecyl-sulfate (SDS) and p-phenylenediamine (PPD) were purchased from Shanghai Sinopharm Chemical Reagent Co., Ltd. (Shanghai, China), and used without further purification. Universal pH test paper (range 1–14) was obtained from Shanghai Sanai Si Reagent Co., Ltd. (Shanghai, China). Aqueous-phase syringe filters (13 mm, 0.45 μm) were purchased from Hefei Baierdi Chemical Technology Co., Ltd. (Hefei, China).

### 2.1. Preparation of Noble Metal Nanomaterials

Preparation of CTAB-Stabilized Gold Nanorods

Gold nanorods were synthesized using the seed-mediated growth method developed by Nikoobakht and El–Sayed [23].

1. Preparation of gold nanoparticle seeds: 103 μL of 1% HAuCl_4_ solution was mixed with 0.1 M CTAB under magnetic stirring at 29 °C until homogeneous. Then, 60 μL of freshly prepared 10 mM NaBH_4_ solution was rapidly added. Stirring was stopped after 2 min, and the solution was allowed to stand.
2. Growth process: 206 μL of 1 w% HAuCl_4_ was mixed with 10 mL of 0.1 M CTAB, followed by sequential addition of 125 μL of 0.008 M AgNO_3_, 100 μL of 2 M HNO_3_, and 60 μL of 0.1 M AA at 29 °C. Finally, 12 μL of the seed solution from step (1) was added.
3. Synthesis of CTAB-stabilized gold nanorods (CTAB-AuNR): The solution from step (2) was mixed thoroughly and left undisturbed in a 28 °C water bath for 12 h to obtain a gold nanorod colloid.

Gold nanoparticles were synthesized using the seed-mediated growth method [24]: 1 mL of 1% (*w*/*w*) HAuCl_4_ solution was added to a 250 mL three-necked flask containing 99 mL of ultrapure water. The mixture was stirred and heated using a temperature-controlled magnetic stirrer. When the mixture began to boil, 4 mL of 1% (*w*/*w*) trisodium citrate was immediately added, and stirring continued for 1 h to obtain a seed solution with a particle size of 15 nm. Subsequently, 25 mL of the seed solution was transferred to a 100 mL three-necked flask and stirred at room temperature. Sequentially, 1 mL of 1% (*w*/*w*) trisodium citrate, 1 mL of 1% (*w*/*w*) PVP solution, and 20 mL of 2.5 mmol/L hydroxylamine hydrochloride were added. While stirring, 20 mL of 2.5 mmol/L HAuCl_4_ solution was added dropwise at a rate of 1 mL/min using a microsyringe pump. After the addition, the solution was stirred for an additional 0.5 h to obtain PVP-AuNPs.

### 2.2. Instruments and Operation

SERS measurements: Raman spectra were acquired using a Horiba confocal Raman system with a 50× objective lens (NA = 0.5). The excitation wavelength was 633 nm, and the laser power was maintained at approximately 0.77 mW to avoid thermal effects or photodamage, with a high-resolution 1800 g/mm grating. The laser was focused onto the sample through a 50×/0.75 N.A. objective, producing a spot size of approximately 1–2 μm, and the integration time was set to 1 s. All SERS spectra were baseline-corrected using LabSpec V6 software.

Scanning electron microscopy (SEM): SEM images were obtained using an Auriga focused ion beam scanning electron microscope (FIB-SEM).

UV-Vis absorption spectra: UV-Vis absorption spectra of nanomaterials were recorded using a UV-2600 spectrophotometer (Shimadzu, Kyoto, Japan).

Zeta potentials: the zeta potentials were measured by Zeta-check, Microtrac (Haan, Germany).

### 2.3. Construction of the Visualized Single D-SERS Detection Platform

**Scheme 1:**
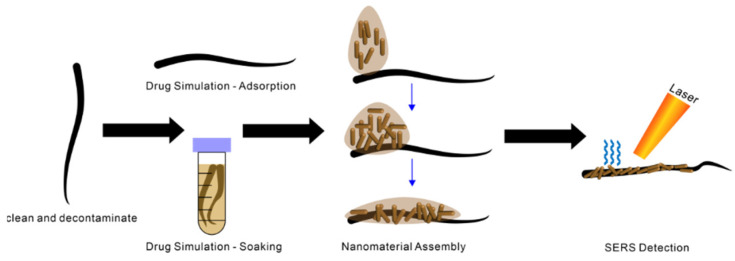
Schematic of nanomaterial assembly and detection on the surface of a single hair strand.

Scheme 1 illustrates the procedure for using a single hair strand as a template for nanomaterial-based target detection. Virgin scalp hair strands were collected from three healthy volunteers who confirmed that they had not dyed, bleached, or permed their hair within the previous six months, the diameters of the hair samples are shown in Table S1, with a maximum of 113 µm and a minimum of 68 µm. Strands were cleaned by sonication for 10 min in 2% (*w*/*v*) sodium SDS, rinsed thoroughly, immersed in ethanol for 5 min, washed with de-ionized water, and air-dried. The hair was then immersed in a solution containing CV or PPD to simulate the enrichment process of drugs or hazardous substances on the hair surface under real-world conditions. After drying, ethanol elution was performed to remove nonspecific multilayer adsorption, ensuring analytical accuracy. A nanomaterial colloid was subsequently dropped onto one end of the hair, enabling spontaneous assembly along the hair surface to form a highly uniform and dense nanomaterial layer. Finally, laser irradiation was applied to the assembled region, enabling highly sensitive Raman signal acquisition of target molecules during the nanomaterial assembly process.

## 3. Results and Discussion

### 3.1. Morphology and Optical Properties of Nanomaterials on Single Hair Surface

#### 3.1.1. UV-Vis Spectral Analysis and SEM Characterization

Figure S1 shows the UV-Vis absorption spectra of CTAB-AuNR and PVP-AuNP. CTAB-AuNR exhibits two absorption peaks at approximately 512 nm and 710 nm, corresponding to the transverse and longitudinal LSPR absorptions of gold nanorods, respectively [23]. The longitudinal LSPR absorption peak position can be tuned by adjusting the aspect ratio of the gold nanorods, enabling SERS enhancement in different wavelength ranges. PVP-AuNP displays a strong, sharp absorption peak near 532 nm, corresponding to the LSPR absorption of gold nanospheres (Figure S1b); the position of this peak is closely related to the size of the nanospheres.

**Figure 1:**
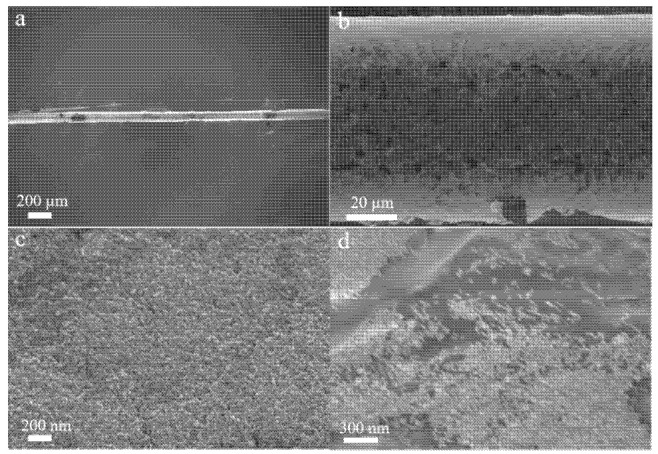
SEM images of CTAB-AuNR on the surface of a single hair strand: (**a**) low-magnification SEM image; (**b**) high-magnification SEM image; (**c**) high-magnification image of CTAB-AuNR on the hair surface; (**d**) high-magnification image of CTAB-AuNR at the hair edge.

Figure 1 presents SEM images of CTAB-AuNR assembled on a single hair strand. The low-magnification SEM image (Figure 1a) shows that CTAB-AuNR forms a highly uniform, dense assembly on the hair surface, covering the entire longitudinal axis. High-magnification SEM images (Figure 1b–d) reveal the microstructure and alignment characteristics of the assemblies. The gold nanorods display a regular, oriented arrangement, with most long axes aligned along the hair axis, indicating that CTAB molecules play a key structural guiding role during assembly. In the edge region of the hair cuticle (Figure 1b), the arrangement of CTAB-AuNR is even more compact and ordered, forming a distinct stacked structure, likely due to the step-like cuticle providing additional confinement effects that promote oriented alignment. Furthermore, Figure 1d clearly shows the morphological features of the gold nanorods: smooth surfaces, uniform diameters (approximately 22 nm), and lengths (approximately 68 nm), with an aspect ratio of approximately 3.1, consistent with that shown in Figure S1a. SEM characterization confirms that CTAB-AuNR forms highly ordered, uniformly oriented assemblies on single hair strands, providing a solid morphological foundation for high-performance, homogeneous, and stable hair-based SERS substrates.

Figure S2 shows SEM images of gold nanoparticles prepared with PVP as a capping agent and assembled on a single hair strand. Images at different magnifications demonstrate that the gold nanoparticles are densely and uniformly distributed (Figure S2a–c), forming a compact nanoparticle film. This closely packed structure supports ultrasensitive detection of trace analytes. Additionally, the high loading of gold nanoparticles on the hair surface (Figure S2d) demonstrates the strong affinity between PVP-capped gold nanoparticles and hair, indicating the broad applicability of hair as a template for constructing SERS substrates.

#### 3.1.2. Mechanistic Investigation of the Confined Assembly of CTAB-AuNR on Single Hair Surface

Human hair is a highly ordered keratin-based biomaterial whose multilayered structure, chemical bonding network, and dynamic growth characteristics provide both functionality and application potential. The hair cuticle comprises overlapping scale-like structures, while the cortex is composed of densely packed keratin fiber bundles [14]. These natural hierarchical structures can act as templates to guide the ordered assembly of nanoparticles. The cuticle layer is arranged in a shingle-like fashion, forming micron-scale grooves and nanoscale gaps (approximately 100 nm in width) that constitute multiscale channels. Under specific pH conditions, the hair surface carries a negative charge due to deprotonation of carboxyl groups, while CTAB-AuNR surfaces are positively charged, as shown in Figure S3, enabling attachment via electrostatic attraction. However, PVP-AuNPs, which have negatively charged surfaces (Figure S3), can also assemble effectively on hair (Figure S2), indicating that directional assembly of nanoparticle colloids on hair is not solely governed by electrostatic interactions.

When the nanoparticle colloid is dropped onto a single hair strand, it rapidly ascends along cuticle gaps under capillary forces, transporting nanoparticles toward the distal end. Upon contact, the colloid climbs swiftly along these grooves, delivering gold nanorods to distal regions through capillary action.

According to the Laplace equation [25], the capillary rise height $h\;=\;\frac{2\gamma \cos\theta }{\rho \mathrm{gr}}$, where γ is the surface tension of the colloid (CTAB reduces γ to ~40 mN/m), r is the effective radius of the groove (~50 nm), and the theoretical capillary rise height can reach several centimeters.

Furthermore, as the colloid ascends along the hair, the solvent gradually evaporates, resulting in a local increase in nanoparticle concentration. This concentration gradient further drives particle enrichment at the liquid–gas interface, forming a dynamic assembly front. The essence of nanoparticle assembly along hair is that capillary transport provides the driving force, electrostatic attraction enables initial adsorption, and the combined influence of surfactant orientation and groove confinement directs the ordered arrangement. By adjusting colloid properties (CTAB concentration, ionic strength) and environmental factors (evaporation rate, pH), the morphology of the nanostructures can be precisely controlled, offering new strategies for developing hair-based photonic devices and flexible sensors.

### 3.2. Mechanistic Analysis of Active Entry of Target Molecules into Nanoparticle Gaps

**Figure 2:**
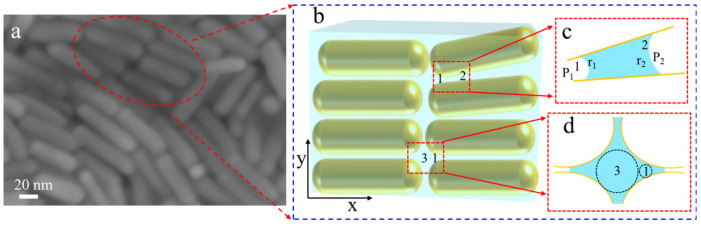
Schematic illustration of target solution migration into smaller gaps in the nanocapillary pump model. (**a**) SEM image of AuNRs; (**b**) Schematic diagram of the AuNR film, with 1, 2, and 3 indicating small gaps between nanorods; (**c**) Local magnification of the gap between two nanorods; (**d**) Local magnification of the gaps among multiple nanorods. r_1_ and r_2_ represent the radii of curvature at different gaps, and P_1_ and P_2_ denote the pressures at different gaps.

In this experimental system, the principle underlying single D-SERS detection during assembly was further analyzed. The nanostructures formed by assembled gold nanorods can be regarded as nano-capillary pump structures composed of closely stacked nanorods, as shown in Figure 2a. Nanoscale gaps with various structural configurations form between adjacent nanoparticles. The force analysis for different gap geometries is presented in Figure 2b. When two nanorods create the gap structure depicted in Figure 2b, the movement of solution within the nanoscale gap can be described by the Young–Laplace equation [26]:

(1) $$\Delta P\;=\frac{\;2\gamma }{r}$$

where ΔP is the additional pressure in the system, γ is the surface tension of the solution, and r is the radius of curvature of the meniscus formed in the nanoscale gap. Owing to the hydrophilicity of gold nanorods, a concave meniscus forms within the nanoscale gap [27]:

(2) $$P_{1}\;=\;\left(P_{1}-P_{e}\right)\;+{\;P}_{e}\;=\;\Delta P_{1}\;+\;P_{e}=-\frac{2\gamma }{r_{1}}\;+{\;P}_{e},$$

(3) $$P_{2}=\left(P_{2}-P_{e}\right)+{\;P}_{e}=\Delta P_{2}+P_{e}=-\frac{2\gamma }{r_{2}}+P_{e},$$

(4) $$\Delta P_{12}=P_{1}-P_{2}=2\gamma \times \left(\frac{1}{r_{2}}-\frac{1}{r_{1}}\right).$$

As shown in Figure 2b, r_2_ > r_1_, ΔP_12_ < 0, indicating that the aqueous solution pressure at position 2 is greater than at position 1, causing the solution to flow from position 2 to position 1. When stacked gold nanorods form the structure shown in Figure 2d, the pressures at different positions (positions 1 and 3 in the figure) can be calculated according to Equations (5) and (6):

(5) $$P_{L3}\;={\;P}_{0}-\Delta P_{3}\;=\;P_{0}-\frac{2\gamma }{r_{3}},$$

(6) $$P_{L1}=P_{0}-\Delta P_{1}=P_{0}-\frac{2\gamma }{r_{1}}.$$

As the radius of curvature r_3_ > r_1_, the internal fluid pressure at position 3 is greater than at position 1 (P_L3_ > P_L2_), and the target solution flows from position 3 into the smaller gap at position 1. In summary, the target solution consistently moves from regions of higher pressure to lower pressure—specifically into smaller gap regions—where the solvent carries analytes into the optimal hot-spot regions. Thus, within the nanoscale gaps, the solution migrates from larger to smaller gaps until complete evaporation. Throughout this process, target molecules follow the solvent’s path, continuously entering the hot-spot regions. This provides the physical basis for highly sensitive SERS detection.

### 3.3. SERS Performance Characterization of the Single D-SERS Detection Platform

**Figure 3:**
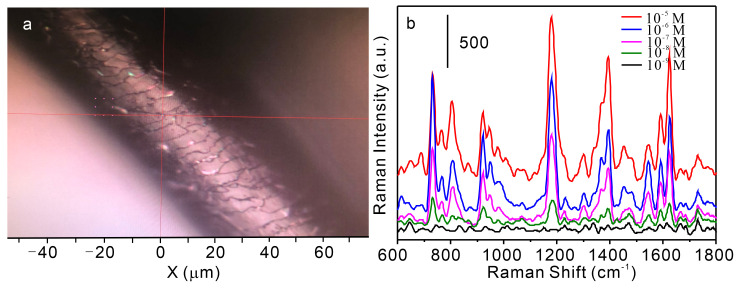
(**a**) Optical photograph of a single hair strand with CTAB-AuNR. (**b**) Detection of various CV concentrations using a single hair strand with CTAB-AuNR.

Figure 3a shows an optical microscopy image of a single hair strand assembled with CTAB-AuNR, captured using the Raman spectrometer’s optical microscope. The surface morphology of the hair is clearly visible, with bright scattering spots. Figure 3b presents the SERS spectra of CV at different concentrations, obtained using the CTAB-AuNR-assembled hair as the SERS substrate. The main characteristic peaks of CV appear at 1620 cm^−1^ (C–C stretching), 1587 cm^−1^ (C–C stretching), 1371 cm^−1^ (N-phenyl ring C stretching), 1178 cm^−1^ (C–H in-plane bending), and 912 cm^−1^ (phenyl ring breathing vibration) [26], consistent with literature reports. As the CV concentration decreases, the intensity of its characteristic peaks gradually declines, indicating that the CTAB-AuNR-assembled hair substrate delivers a reliable SERS response to CV. Notably, even at a concentration of 10^−9^ M, the characteristic peaks of CV remain clearly distinguishable, confirming that the CTAB-AuNR-assembled hair substrate offers high SERS detection sensitivity and holds strong potential for ultrasensitive trace analyte detection.

Figure S4 further demonstrates the reproducibility of SERS detection for CV molecules at varying concentrations using the CTAB-AuNR-assembled SERS substrate on a single hair strand. Figure S4a shows the SERS spectrum of CTAB-AuNR assembled on blank hair, where no distinct characteristic peaks are observed, indicating that the CTAB-AuNR itself contributes negligibly to the SERS signal and does not interfere with target detection. As the CV concentration decreases, the intensity of each characteristic peak gradually weakens; however, even at concentrations as low as 10^−9^ M, these characteristic peaks remain clearly discernible, confirming the high sensitivity of the CTAB-AuNR-assembled hair SERS substrate. To further investigate the reproducibility both between and within substrates, we collected SERS data for CV detection using 20 hair samples from three volunteers and calculated the relative standard deviation (RSD). As shown in Table S2, the RSD of SERS intensity at 1620 cm^−1^ for 10 randomly selected detection points on each hair sample was less than 10%. The overall RSD calculated from all 200 data points was 19.19%, which is below 20%. Additionally, three random detection results from each of the 20 hair samples were selected to generate Figure S5, which shows an RSD of 18.86%. These results indicate that the detection reproducibility meets the requirements, both for individual hair samples and between different samples.

Figure S6 presents the SERS detection results after a single hair strand was immersed in 10^−6^ M CV solution, dried, and washed with distilled water 1–4 times, followed by CTAB-AuNR assembly on the hair surface. With increasing washing cycles, the overall intensity of the SERS spectra gradually decreases, but the positions and relative intensities of the characteristic peaks remain unchanged. The peaks at 1620 cm^−1^ and 1587 cm^−1^ originate from the C–C stretching and anomalous mode vibrations of the benzene ring in CV, while the peaks at 1371 cm^−1^ and 1178 cm^−1^ correspond to the C–N stretching and C–H in-plane bending vibrations of the N-phenyl group. Even after four washes, these characteristic peaks remain clearly identifiable, indicating that hair has a strong adsorption capacity for CV molecules, which facilitates enrichment and enables ultrasensitive detection. Moreover, the washing process does not introduce interference peaks or cause significant shifts in peak positions, demonstrating the excellent selectivity and stability of the CTAB-AuNR-assembled hair SERS substrate. Figure S7 shows the SERS spectra of CV at different concentrations detected by PVP-AuNPs assembled on a single hair surface, demonstrating the universality of this method. This substrate, therefore, shows strong potential for rapid, on-site detection of real samples.

**Figure 4:**
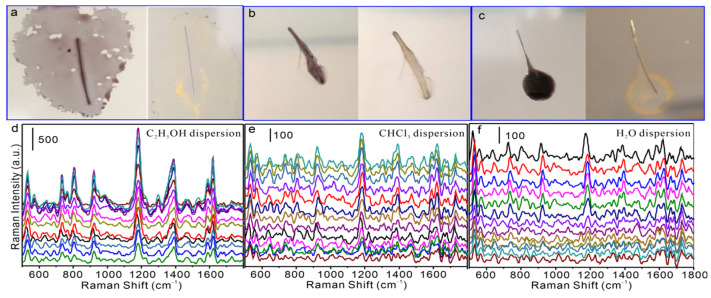
Detection of 10^−6^ M CV on hair surfaces using CTAB-AuNR assembled with different solvent dispersants. Optical image of a single hair strand assembled with CTAB-AuNR using as dispersant (**a**) ethanol (Left side during assembly process, right side after drying); (**b**) chloroform (Left side during assembly process, right side after drying); (**c**) water (left side during assembly process, right side after drying). Time-resolved SERS spectra of CV detected by CTAB-AuNR assembled using as dispersant (**d**) ethanol; (**e**) chloroform; (**f**) water.

Figure 4 presents the SERS spectra obtained from CTAB-AuNR assemblies prepared using different dispersants. A single hair strand was immersed in a 10^−6^ M CV solution, dried, and then assembled with CTAB-AuNR dispersed in ethanol, chloroform, or water. The optical images of these assemblies under different dispersant conditions reveal clear morphological differences (Figure 4a–c), attributable to the distinct surface tensions of the three solvents, which influence wetting and spreading on the hair surface. Ethanol, with the lowest surface tension (~22 mN/m), exhibits the strongest wetting and spreading capability, enabling uniform dispersion and close packing of gold nanorods (Figure 4a) to form a well-ordered assembly structure. This optimal morphology promotes the generation of numerous “hot spot” regions, producing strong electromagnetic field enhancements [9] and delivering the highest SERS performance (Figure 4d). Chloroform, with a surface tension (~27 mN/m) higher than ethanol but lower than water, achieves intermediate spreading and assembly quality on the hair surface (Figure 4b). Accordingly, the SERS spectral intensity obtained with chloroform as the dispersant is lower than that with ethanol but higher than that with water (Figure 4e). Water, having the highest surface tension (~73 mN/m), exhibits the weakest wetting and spreading ability on the hair surface, resulting in lower assembly density and uniformity of gold nanorods (Figure 4c). Its high dielectric constant may also reduce electrostatic repulsion between gold nanorods, promoting a degree of aggregation; thus, the SERS spectral intensity achieved with water is the lowest (Figure 4f). The surface tension of the dispersant markedly influences its wetting and spreading behavior on the hair surface, thereby determining the assembly morphology of CTAB-AuNR and the resulting SERS enhancement. Selecting dispersants with low surface tension, such as ethanol, is advantageous for forming highly uniform and densely ordered assemblies, thereby maximizing SERS detection sensitivity.

Since hair is primarily composed of protein (keratin), it is also essential to investigate the stability of the detection platform after nanoparticle assembly. We stored the hair samples assembled with nanomaterials for two weeks and conducted comparative tests. According to the experimental results, the RSD values of the substrates before and after storage were 10.74% and 9.44%, respectively, as shown in Figure S8, indicating that the reproducibility of the substrates remained essentially unchanged after storage. However, the average SERS signal intensity of CV on the platform stored for two weeks decreased by approximately 1.8 times, although no significant morphological changes in the substrate were observed. This may be due to the relaxation of the hydrophobic chains of the surfactant on the substrate surface during storage in air, making it more difficult for target molecules to approach the gold surface, thereby reducing the amount of chemisorption and the occupancy rate of “hot spots.” Alternatively, slight swelling of the hair keratin due to moisture absorption may slightly increase the gaps between nanomaterials, weakening electromagnetic coupling; since this process occurs relatively uniformly along the hair fiber, the RSD remains largely unchanged. However, the current storage duration is not sufficiently long, and in future studies, we will further strengthen the data analysis on substrate stability.

### 3.4. Application of Nanomaterials on Single Hair Surface for Real Sample Detection

Figure S9 presents the Raman spectrum of solid PPD, characterized by a series of distinct and sharp peaks. The most intense peak at 1610 cm^−1^ corresponds to the C–C stretching vibration of the benzene ring. Peaks at 1280 cm^−1^ and 1235 cm^−1^ are assigned to C–N stretching and NH_2_ wagging vibrations, respectively. Additional peaks in the 800–1200 cm^−1^ range arise from in-plane and out-of-plane C–H bending vibrations on the benzene ring [28]. The positions and relative intensities of these peaks reflect the molecular structure and vibrational modes of PPD, providing critical spectral fingerprints for sensitive detection via SERS.

Figure S10 presents the SERS spectra of PPD at different concentrations detected using CTAB-AuNRs. Significant enhancement effects are still evident at a concentration of 10^−6^ M, with characteristic peaks at 1200 cm^−1^, 1420 cm^−1^, and 1610 cm^−1^ remaining clearly distinguishable, underscoring the excellent performance of SERS in trace detection. Compared with conventional Raman spectra, the SERS spectra show markedly increased signal intensity, highlighting the broad application potential of this technique in analytical chemistry. Despite the substantial signal enhancement, the positions of the characteristic peaks remain unchanged, indicating that the SERS mechanism does not alter the molecular vibrational modes of PPD. This finding not only offers a new technical approach for detecting PPD but also supports safety assessments of harmful hair dyes.

**Figure 5:**
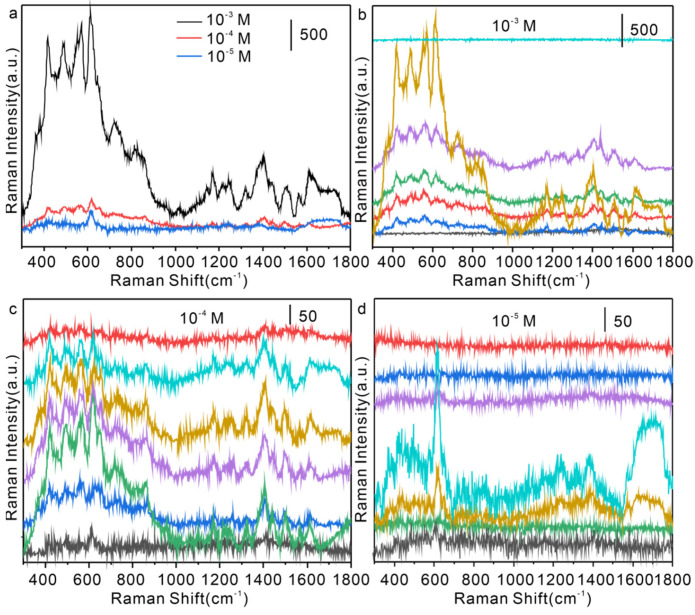
SERS spectra of PPD detected on hair surfaces using the single D-SERS detection platform. (**a**) SERS spectra of PPD at high sensitivity; (**b**) 10^−3^ M PPD; (**c**) 10^−4^ M PPD; (**d**) 10^−5^ M PPD.

Figure 5 shows the SERS spectra of PPD detected using the single D-SERS platform on hair surfaces. In the experiment, single hair strands were immersed in PPD solutions of varying concentrations for 24 h, dried, and then subjected to gold nanorod self-assembly, during which single D-SERS detection was performed. Figure 5a presents the SERS spectra of hair treated with 10^−3^, 10^−4^, and 10^−5^ M PPD solutions, where the C–C stretching vibration of the benzene ring at 1610 cm^−1^ is prominent. As the PPD concentration decreases, the intensity of this peak gradually diminishes, demonstrating clear concentration dependence.

Figure 5b–d illustrate the spatial distribution of SERS signals at various positions along a single hair strand under different concentrations. At 10^−3^ M, high-intensity and consistent Raman signals are observed at all points on the hair surface, indicating a uniform distribution of target molecules. At 10^−4^ M, although the signal intensity is reduced, the characteristic peaks remain discernible with good reproducibility. At 10^−5^ M, some measurement points yield signals approaching the noise level, yet characteristic peaks of PPD are still detected in certain regions, reflecting the high sensitivity and local enrichment capability of this method for trace detection.

These experimental results validate the feasibility of using pristine single hairs as SERS substrates. Nevertheless, we note that real consumer-dyed hairs involve multicomponent colorants, after-dye care products, and variable aging histories, all of which may affect adsorption and spectral interpretation. A systematic evaluation on large, categorized sets of such samples is beyond the scope of the present proof-of-concept work but will be pursued in future studies.

## 4. Conclusions

In this study, a single-hair in situ D-SERS detection platform was developed using a natural hair template and confined assembly, enabling the oriented and dense arrangement of CTAB-AuNR and PVP-AuNP within the cuticle grooves of hair. This approach markedly improved the substrate’s uniformity and stability. UV-Vis and SEM analyses confirmed the plasmonic and morphological characteristics of the gold nanorods and spheres. Furthermore, the use of different dispersants (with ethanol outperforming chloroform and water) enhanced wetting and spreading, thereby increasing hotspot density. The platform achieved reliable detection of CV at concentrations as low as 10^−9^ M, with minimal blank interference and stable peak positions and relative intensities after washing. Meanwhile, signal stability can be maintained within a certain storage period. In real sample systems, the platform enabled concentration-dependent and spatially resolved detection of PPD on hair strands in the range 10^−3^–10^−5^ M, demonstrating strong potential for rapid, portable, and cost-effective applications. A current limitation of this study is the lack of comprehensive tests on commercially dyed or environmentally exposed hair strands, whose chemical heterogeneity demands a larger sample pool and more refined spectral deconvolution. We are now designing an expanded study to address these real-world scenarios and further demonstrate the platform’s practical utility.

## Data Availability

The raw data supporting the conclusions of this article will be made available by the authors on request.

## Supplementary Materials

The following supporting information can be downloaded at https://www.mdpi.com/article/10.3390/nano15201557/s1: Table S1: Average Diameter of Hair Samples from Three Volunteers; Figure S1: SEM and UV-Vis spectrophotometric characterization of noble metal nanomaterials: (a) CTAB-AuNR, (b) PVP-AuNP; Figure S2: SEM images of PVP-AuNP assembled on the surface of a single hair strand: (a) Low-magnification SEM image, (b) higher-magnification SEM image, (c) further magnified SEM image, and (d) high-magnification SEM image of PVP-AuNP on the hair surface; Figure S3: Zeta potential data of CTAB-AuNRs and PVP-AuNPs; Figure S4: (a) Time-resolved SERS spectra of blank hair using a single hair strand assembled with CTAB-AuNRs; (b–f) Time-resolved SERS spectra of CV at concentrations ranging from 10^−5^ M to 10^−9^ M; Table S2: Relative standard deviations (RSD) of SERS intensity at 1620 cm^−1^ for 10 randomly selected detection points on each of the 20 hair samples; Figure S5: SERS intensity of the characteristic peak at 1620 cm^−1^ for three randomly selected detection points on each of the 20 hair samples; Figure S6: Time-resolved SERS spectra of 10^−6^ M CV on hair surfaces assembled with CTAB-AuNRs after washing (a) once, (b) twice, (c) thrice, and (d) four times; Figure S7: Detection of CV at different concentrations using PVP-AuNPs; Figure S8. SERS intensity of the characteristic peak of 10^−6^M CV at 1620 cm^−1^ detected by the detection platform (30 measurements). (a) Current sample; (b) sample after 2 weeks of storage; Figure S9: Raman spectrum of solid PPD; Figure S10: SERS spectra of PPD at different concentrations detected by CTAB-AuNR.

## Abbreviations

The following abbreviations are used in this manuscript:

NaBH_4_	Sodium borohydride
NaOH	Sodium hydroxide
AgNO_3_	Silver nitrate
HNO_3_	Nitric acid
PVP	Polyvinylpyrrolidone
H_2_O_2_	Hydrogen peroxide
AA	Ascorbic acid
PPD	p-Phenylenediamine
SERS	Surface-enhanced raman spectroscopy
D-SERS	Dynamic-SERS
LSPR	Localized surface plasmon resonance
UV-Vis	Ultraviolet-visible
SEM	Scanning electron microscopy
RSD	relative standard deviation
