# Supplementary material for: Hair-Template Confinement Assembly of Nanomaterials Enables a Robust Single-Hair Surface-Enhanced Raman Spectrocopy Platform for Trace Analysis
Source: Nanomaterials (Basel). 2025 Oct 13;15(20):1557. doi: 10.3390/nano15201557 (PMC12566538; doi:10.3390/nano15201557)
Supplement: Supplementary file 1 [file nanomaterials-15-01557-s001.zip › nanomaterials-3869524-supplementary.pdf]

Supplementary Materials

Hair-Template Confinement Assembly of Nanomaterials Enables a Robust Single-Hair Surface-Enhanced Raman Spectrocopy Platform for Trace Analysis

Miao Qin [1,*], Siyu Chen [2], Tao Xie [3], Mingwen Ma [1] and Cong Wang [1]

[1] Key Laboratory of Spin Electron and Nanomaterials of Anhui Higher Education Institutes, School of Chemistry and Chemical Engineering, Suzhou University, Suzhou 234000, China; mamingwen163@163.com (M.M.); congwang@ahszu.edu.cn (C.W.)
[2] Information Materials and Intelligent Sensing Laboratory of Anhui Province, Anhui University, Hefei 230039, China; 23068@ahu.edu.cn
[3] Key Laboratory of Conservation and Utilization of Dabie Mountain Characteristic Biological Resources, West Anhui University, Lu'an 237012, China; xt2020@mail.ustc.edu.cn
* Correspondence: mqin@mail.ustc.edu.cn; Tel.: +86-18712111566

Table S1. Average Diameter of Hair Samples from Three Volunteers

Sample / Project	Volunteer 1	Volunteer 2	Volunteer 3
Average diameter	74±6 μm	82±8 μm	105±8 μm

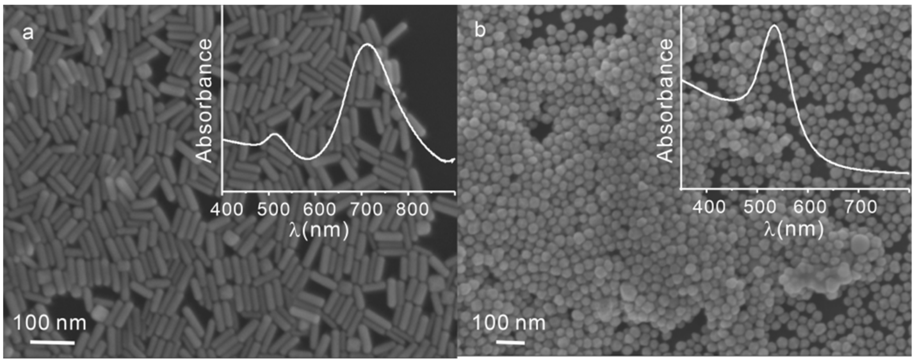

Figure S1. Scanning electron microscopy and UV-Vis spectrophotometric characterization of noble-metal nanomaterials: (a) CTAB-AuNR; (b) PVP-AuNP.

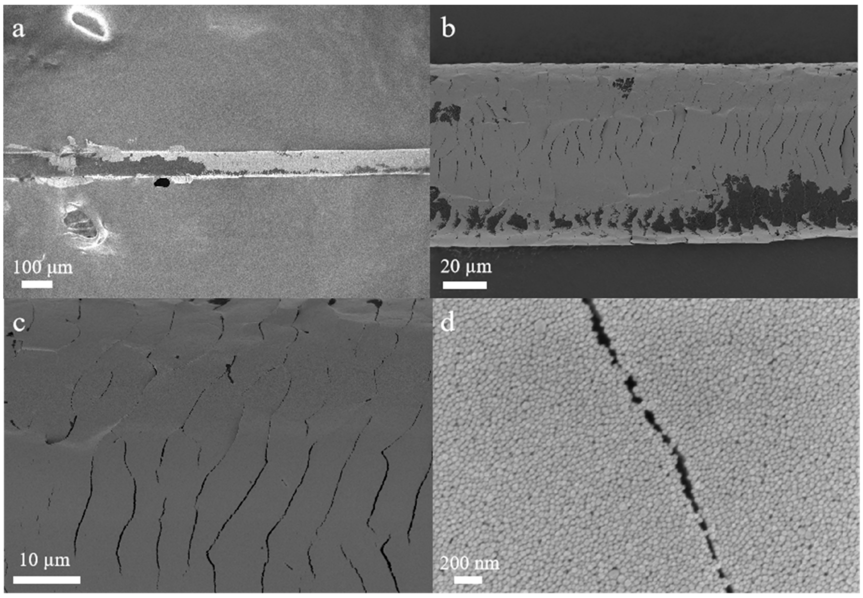

Figure S2. SEM images of PVP-AuNP assembled on the surface of a single hair strand. (a) Low-magnification SEM image; (b) Higher-magnification SEM image; (c) Further magnified SEM image; (d) High-magnification SEM image of PVP-AuNP on the hair surface.

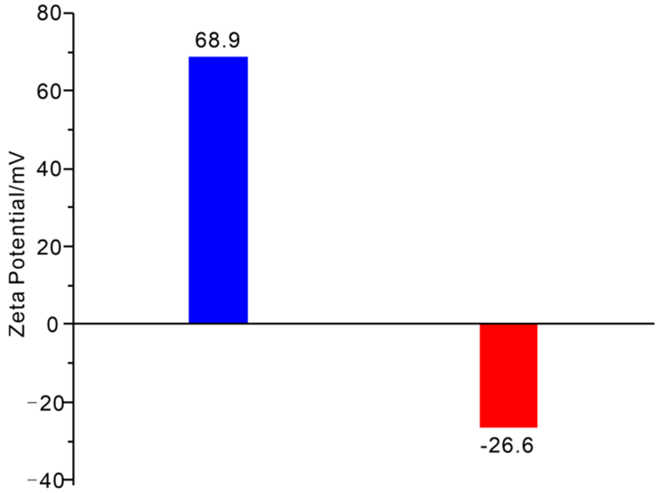

Figure S3. Zeta potential data of CTAB-AuNR and PVP-AuNP.

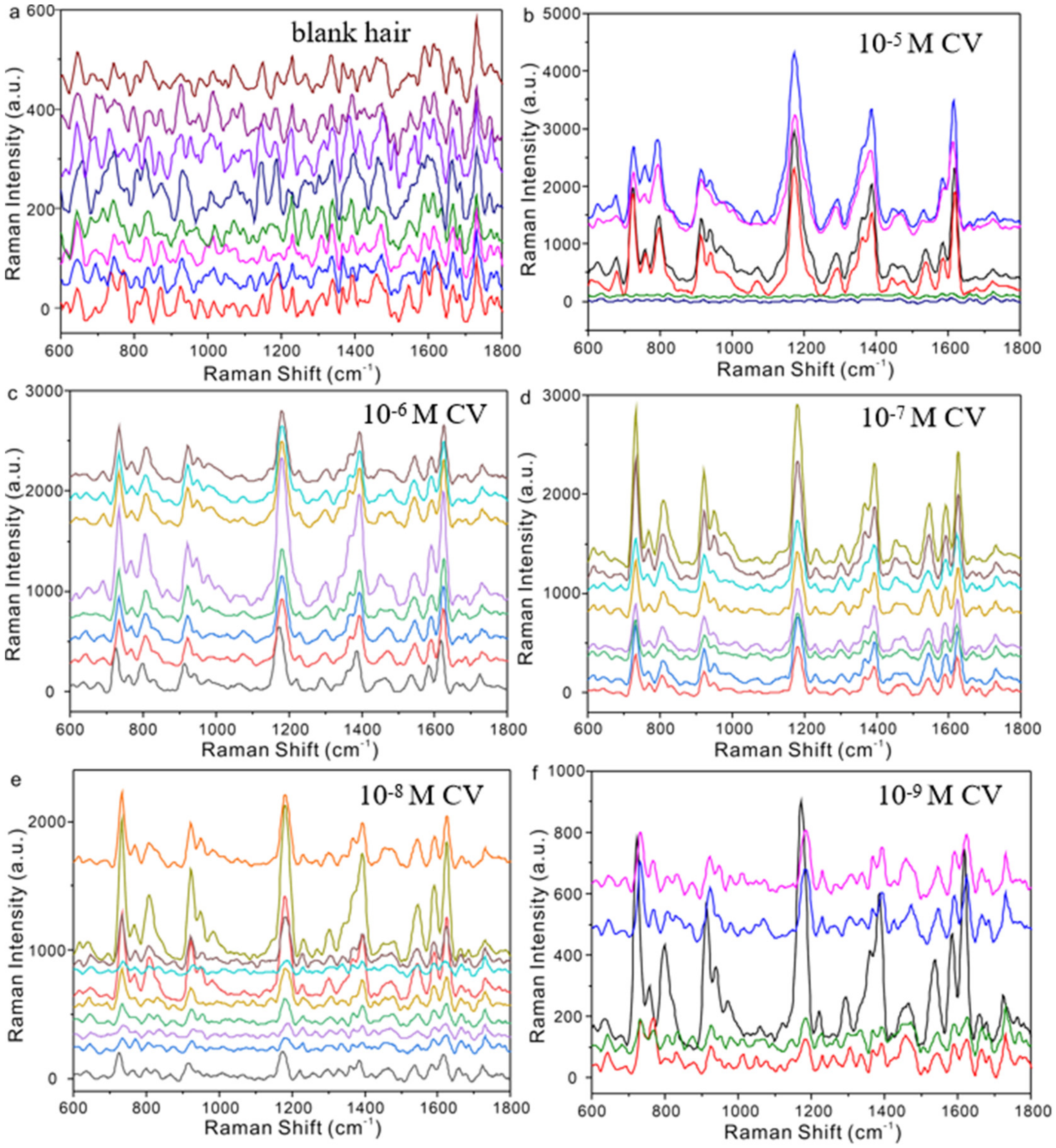

Figure S4. (a) Time-resolved SERS spectra of blank hair using a single hair strand assembled with CTAB-AuNRs; (b–f) Time-resolved SERS spectra of CV at concentrations ranging from 10^{-5} M to 10^{-9} M.

Table S2. Relative standard deviations (RSD) of SERS intensity at 1620 cm^{-1} for 10 randomly selected detection points on each of the 20 hair samples

Sample / point	Sample1	Sample2	Sample3	Sample4	Sample5	Sample6	Sample7	Sample8	Sample9	Sample10
RSD	4.14%	5.35%	7.13%	4.09%	5.80%	6.45%	3.43%	3.21%	4.98%	7.43%
Sample / point	Sample11	Sample12	Sample13	Sample14	Sample15	Sample16	Sample17	Sample18	Sample19	Sample20
RSD	3.16%	3.32%	8.93%	3.05%	3.12%	4.46%	3.86%	3.63%	3.25%	3.04%

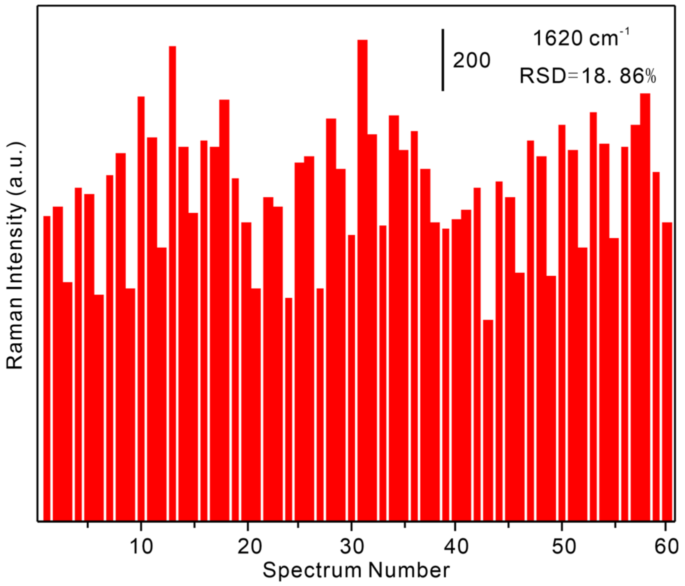

Figure S5. SERS intensity of the characteristic peak at 1620 cm^{-1} for three randomly selected detection points on each of the 20 hair samples

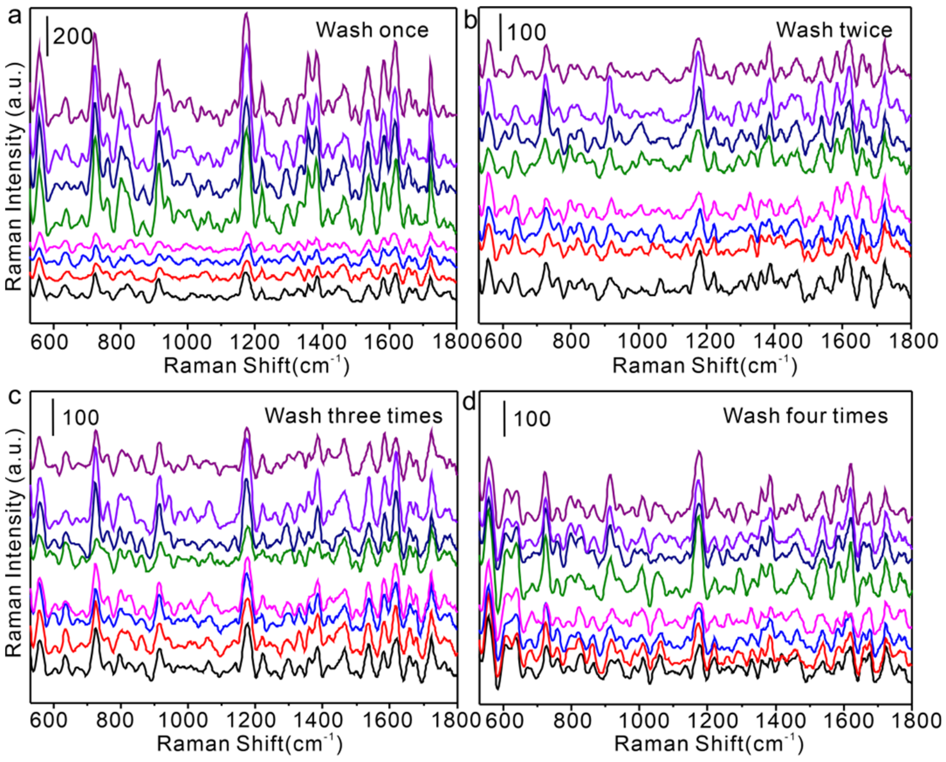

Figure S6. Time-resolved SERS spectra of 10^{-6} M CV on hair surfaces assembled with CTAB-AuNRs after washing (a) once, (b) twice, (c) three times, and (d) four times.

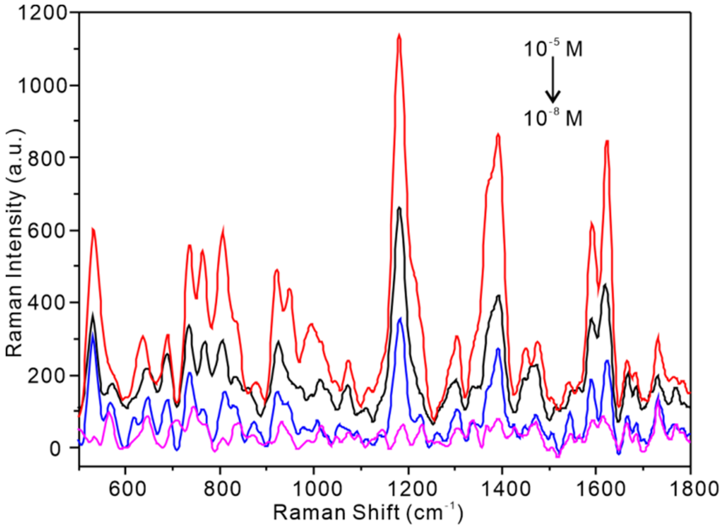

Figure S7. Detection of CV at different concentrations using PVP-AuNPs.

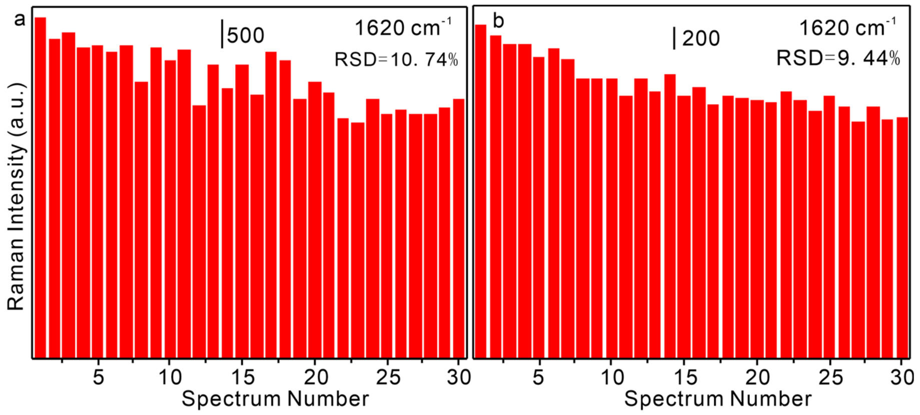

Figure S8. SERS intensity of the characteristic peak of 10^{-8} M CV at 1620 cm^{-1} detected by the detection platform (30 measurements). (a) Current sample; (b) sample after 2 weeks of storage.

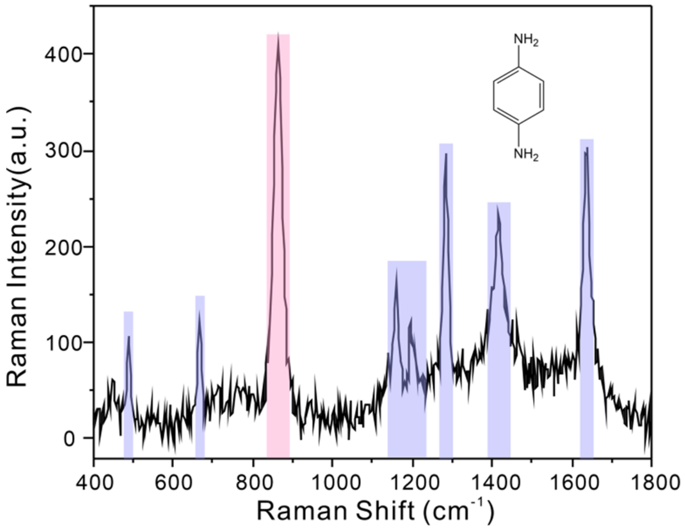

Figure S9. Raman spectrum of solid PPD.

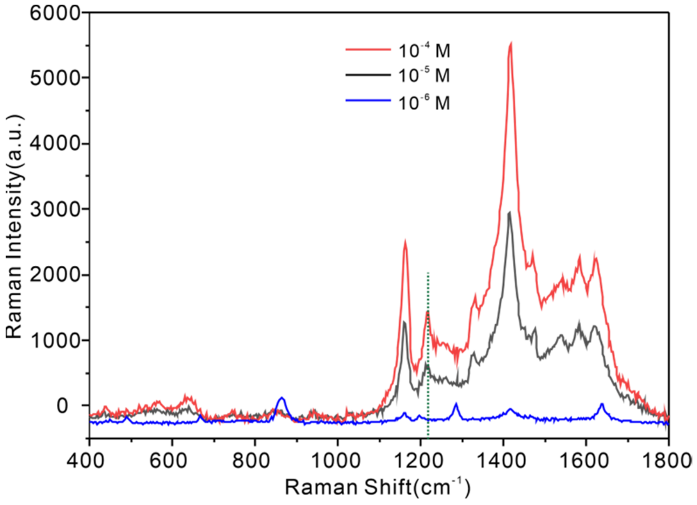

Figure S10. SERS spectra of PPD at different concentrations detected by CTAB-AuNR.
